# Fifteen Cases of “Cerebro-Spinal Meningitis,” and Their Post Mortem Appearances

**Published:** 1867-01

**Authors:** Chas. M. Clark

**Affiliations:** Late Surgeon 39th Ill. Vol. Infantry, and Chief Operating Surgeon 24th Army Corps


### CHICAGO MEDICAL JOURNAL.

VOL. XXIV. JANUARY, 1867. No. I.

### ORIGINAL CONTRIBUTIONS.

*Fifteen Cases of "Cerebro-Spinal Meningitis," and their Post Mortem Appearances.* Presented by CHAS. M. CLARK, M.D., late Surgeon 39th Ill. Vol. Infantry, and Chief Operating Surgeon 24th Army Corps.

During the months of January, February, and March, 1865, a large number of cases came under my notice at the field hospital of the 24th Army Corps, which, at that time, was located near Hutchin's Run, Virginia. I took especial interest in studying the disease, and in making full and thorough examinations after death, which I am sorry to add, was the termination in nearly every case that presented for treatment.

CASE I. Benjamin Hyman, private, Co. F. 11th West Virginia Regiment, American by birth, aged 20 years; admitted to hospital February 20th, 1865. On his admission he was fiercely delirious, and after being placed in bed he would not lie quietly, but kept changing his position from side to side; would often break out into loud exclamations and moans; often starting up with a wild, staring expression of countenance, and, if not forcibly prevented, would get up and saunter down through the aisle of the ward. His pulse was 80; and soon after his entrance, he vomited a large quantity of a dark green matter. The pupils of the eyes were slightly contracted; the tongue moist and normal; skin dry and cold; and the hands and feet were purplish in color. The urine was scanty and very high colored, and the bowels were costive.

He was placed in a warm bath, and after his return to bed a mild cathartic was given and a blister applied to the nape of the neck, together with a sinapism to the plantar surface of each foot. Ten grains of Dover's powder were also administered.

*Progress of case.—Feb'y 21.* Very restless and wild; no sleep during the night; pulse 120, very small and irregular; bowels moved during the night. During the day, petechiæ of various sizes commenced to appear, mostly noticeable on the fore-arms and legs. Quinine and acetate of potash ordered to be given him.

*Feb'y 22.* No perceptible alteration in the symptoms. A catheter was passed and a small quantity of dark urine was drawn off. Quinine and potash continued, with the addition of milk punch every few hours.

No considerable change occurred in the case, with the exception of increasing weakness, until *Feb'y 26th*, when the pupils became dilated and there was a disposition to cough. The delirium at this time was more mild; there was disposition to sleep; and the patient lay comparatively quiet on his bed. The bowels were still inclined to be costive; urine scanty and high-colored; tongue dry through the centre, but not furred or discolored. He had some appetite, but did not wish anything but pie to eat. Continued same treatment.

*Feb'y 27.* Had involuntary dejections from the bowels; urine more free, not so highly-colored, and passed naturally; pulse varied from 80 to 130; lay comparatively quiet and comatose; pupils largely dilated, and the eyes injected. During the day, he commenced throwing back his head, and there was some trembling of the limbs.

*Feb'y 28.* No change. Some vomiting of greenish matter. Some difficulty of deglutition noticed.

*March 1.* Continues apparently the same. Dimness of vision noticed; he is also becoming quite deaf.

He passed along in this state, growing gradually weaker; tongue dry and moist by turns; sub-sultus increasing; pulse gradually failing, until *March 5th*, when he died, at 9 o'clock P.M. Just previous to his death, pustules of acne broke out, most apparent about the face and neck, but some few crops were seen on the fore-arms and legs.

SECTIO CADAV. 17 HOURS AFTER DEATH.

Rigor mortis well marked. Body not emaciated.

*Head.*—On removing the calvaria, the dura mater was found greatly injected with both arterial and venous blood, and somewhat thickened. The arachnoid had a shiny opalescent look, and was noticeably thickened at about the middle and on either side of the longitudinal fissure. On reaching the pia mater, it was found covered with patches of lymph and pus. On removal of the brain from the skull, a large quantity of serum, flaky with pus, escaped from the membranes of the spinal cord, and when the brain was placed on the table, by slight pressure, some six ounces of serum escaped from the membranes and third ventricle. In the spinal canal, three drachms of thick, yellow pus were found. Over the pons varolii, optic tract, and the base of the cerebellum a thick layer of laudable pus was seen, and some was also found between the lobes of the cerebellum. On cutting down through the left hemisphere of the cerebrum to the corpus callosum and opening the lateral ventricle, three drachms of flaky serum were found, and in the anterior and posterior cornua, there was one drachm of thick pus. The right lateral ventricle presented the same appearance. Pus was also found in the third and fourth ventricles. The brain tissue, especially the cortical portion of both cerebrum and cerebellum, was soft and pultaceous and easily broken up. The medullary portion did not seem greatly altered, although the puncta vasculosa were more numerous than is usual.

*Chest.*—The lungs and heart were found normal.

*Abdomen.*—The organs in this division were healthy, and nothing appeared abnormal except the under surface of the liver, which presented a very dark, shining look. The gall-bladder was empty.

CASE II. Gaines Reynolds, private, Co. I., 89th N.Y. Vols.,

American, aged 24 years. This man was admitted to hospital February 7th, 1865, with the following history, forwarded by Surgeon Squires at the time:—"Man was taken sick January 17th with chill; severe pain in head, back, extremities, and the surface of the body generally. Vomiting was an early and persistent symptom. Blue pills and a saline cathartic were administered in the first place, and three blisters were used: one to the back of the neck, and one to either calf of leg.

A threatening collapse occurred about twelve hours from the seizure, in which the patient came near dying. Stimulants were freely used and heat applied; reaction finally came on. The bowels were moved by the physic, but did not move again in a week's time. He vomited every day. His chief complaint was pain in his head, back, bones, flesh, and all over. His eyes were red at first, and discharged a little; his hearing was very acute, and the least noise greatly disturbed him. There was great sluggishness in urinating. The head was disposed to be retracted. He was rational all the time; no convulsions; no delirium; no paralysis. The pains gradually left him, and he was sent to Field Hospital."

The symptoms of this man, on entering hospital, were:—Slight pyrexia; eyes injected, looking like a case of acute conjunctivitis; pupils greatly enlarged; very restless in body, but evinced no derangement of mind. He was not disposed to talk much, but would answer questions correctly and fully. No delirium throughout. His tongue was thickly coated; and, during his stay in hospital, he had several involuntary discharges from the bowels. His kidneys acted freely and naturally, no catheter being necessary, and there was no especial deposit noticeable in the urine voided. The heat about the head was greatly increased, and there was a constant desire to keep the head thrown back. His appetite was moderate; did not desire food, but would eat his toast and drink tea or coffee at the usual hour. He was placed upon quinine and nit. potass., together with milk punch. Anodynes were given when necessary. and cold was constantly applied to the head.

The case thus passed on, without change, until 6 o'clock P.M., February 13th, when he died comatose.

SECTIO CADAV. 2 P.M., FEBRUARY 14TH.

Rigor mortis not well marked. The body not emaciated.

*Head.*—The first appearance on removing the skull cap, is extensive congestion of the dura mater; some slight adhesions between the dura mater and arachnoid. On reaching the pia mater, there was quite a flow of serum (flocculent), and on each side of the longitudinal sinus there was an extensive exudation of pus. On removing the brain, there was found, at the time the section was made, a considerable flow from the meninges. There was a thick layer of pus over the whole of the medulla oblongata. On opening the membrane near the optic commissure, there were found two ounces of serum. The lateral ventricles were found full of serum, of a turbid character. The fourth ventricle was also found full. The brain tissue was softened throughout its whole extent. Upon examination of the cervical portion of the cord, some pus was found, and the cord was softened.

*Chest.*—The left lung was covered with false membrane throughout its whole extent, and was firmly bound down posteriorly; the pleura was also firmly adherent to the pericardium. The lung was full of a tubercular deposit, of the miliary character, together with some of the other forms; the right lung was normal. Great hypertrophy of the pericardium on the left side; no effusion within it. There was an abnormal fatty deposit over the whole anterior surface of the heart.

*Abdomen.*—The substance of the liver was engorged; the gall-bladder was greatly distended; spleen normal; kidneys of usual character.

*Intestines.*—Omentum thickened and somewhat diseased; transverse colon contracted to one-third its normal size; mesentery very fatty; ileum considerably congested, but no ulcerations found, its coats were very thin. A lumbricoid worm was found in the jejunum. Descending colon thickened and constricted; bladder distended with urine.

CASE III. Emory Wells, private, Co. D., 39th Ill. Vols., American, aged 25 years. This man entered hospital at Richmond, Va., June 14th, 1865, at 10 o'clock A.M. At the time of his entrance, he was comatose; pupils largely dilated; tongue moist, but not furred; pulse 140 to 150; great disposition to tonic spasms; keeping the head thrown back; no apparent delirium; urine and fæces passed off freely and naturally; the eyes were congested and suffused; and respiration was very rapid.

Dr. James Crozier, Assistant-Surgeon in charge of the regiment, stated that the man was apparently well the day before his entrance to hospital, although since his enlistment into service he had never been able to perform the full duties of a soldier. No change occurred in the condition of this man, and no treatment was given him, as it was apparent that he must soon die. He died at 11:30 P.M., June 15th, 1865, just 13 hours and 30 minutes after his entrance.

AUTOPSY HELD AT 12 M., JUNE 16TH, 1865.

Body emaciated; rigor mortis not well marked.

*Head.*—On removal of the skull cap, the dura mater was found greatly injected with blood; no especial change was manifest in the arachnoid, but over the entire surface of the pia mater there was a large deposition of thick, yellowish pus; the brain tissue proper, was found infiltrated, greatly congested, and very much softened—a slight stream of water would wash it away; pus was found distributed in patches over the whole brain, and in its sulci underneath the pia mater; the right and left ventricles were full of bloody serum, which was flaky with pus corpuscles; the third and fourth ventricles presented the same appearance.

*Chest.*—Lungs and heart were found normal.

*Abdomen.*—All of the contained organs were in a healthy condition except the kidneys, which showed a little fatty degeneration.

I have neglected to mention that the medulla oblongata and the cervical portion of the spinal cord were infiltrated with pus.

CASE IV. Dennis Brow, private, Co. M., 4th Mass. Cav., aged 26 years. This man entered hospital February 19th, 1865. When first seen, he was suffering with a severe chill; he was delirious, and suffered considerable dyspnœa; his tongue was red, with a brown centre; the pupils were natural; skin dry and moist; pulse scarcely perceptible at the wrist, very frequent and irregular, 120 to 150; eyes red and highly injected; diarrhœa, with involuntary discharges from the bowels; respiration short and quick; respiratory murmur clear and distinct; no cough; very restless, with some tenderness over the abdomen; purpura hemorrhagica appeared in spots over the whole surface of the body; is indisposed to talk, and it is with great difficulty that he can be made to answer questions.

This patient presented (according to the history furnished by the surgeon of his regiment) every appearance of enjoying good health previous to this sickness, which attacked him the day previous to his entrance to hospital.

*Treatment.*—He was put in a warm bath, containing mustard; sinapisms to the epigastrium, inside of the thighs and ankles; a saline cathartic was given, and quinine and Dover's powder were ordered during the night.

*Progress of the case.*—On visiting him next morning, I found him a little more rational, yet there was wandering of the mind and disposition to talk; there was an increase of the purpura over the body; breathing was more natural; pulse imperceptible at the wrist; skin cool and moist; had several involuntary evacuations during the night; bowels still tender; vomited occasionally a greenish matter, and could retain nothing on the stomach; there was a puffy condition of the face and neck; the pupils were enlarged; and the whole surface of the skin was hyperæsthetic. The patient gradually sunk, and died at 2:30 o'clock P.M.

AUTOPSY, 11 O'CLOCK A.M., FEBRUARY 21ST, 1865.

*Brain.*—The dura mater presented petechial discoloration over the whole of the superior surface, considerable effusion underneath it; arachnoid somewhat thickened; pia mater extensively injected with blood, with here and there a thick purplish streak, pus found in patches over its whole extent; lateral ventricles full of bloody serum; thick pus over optic tract; the cerebellum presented the same appearances; there was pus in the fourth ventricle, and in the spinal canal.

*Chest.*—Right lung closely adherent to the pleura costalis; and the pleura over both lungs presents a mottled appearance; lung tissue healthy. On opening the pericardium, found it greatly congested and spotted; thick purple streaks of congestion are numerous. The pericardium contained two ounces of serum, largely mixed with pus. The heart itself is mottled and is covered with large patches of pus, especially around the sinuses of the aorta. The muscular tissue of the heart is thickened and condensed, cutting like cartilage. Left ventricle contained one ounce of thin bloody serum.

*Abdomen.*—The liver, both externally and internally, presents patches of congestion; a small ulcer was found in the left lobe; substance of liver greatly softened. Spleen one-third larger than normal. Kidneys normal.

*Intestines.*—The whole tract was covered with the hemorrhagic spots; otherwise it was healthy. The stomach was largely distended with gas. Bladder found full of urine.

CASE V. William Statlen, American, aged 21 years, private, Co. C., 15th West Va. Vols. Admitted February 26th, 1865. Symptoms on entrance:—High fever; pulse 120; considerably delirious and extremely restless; pupils contracted; he could not be aroused or made to understand anything; petechial spots were present over the whole surface of the body, but were most marked over the chest and abdomen; had attacks of vomiting every half-hour or so, when he would throw up small quantities of greenish fluid.

*Treatment.*—A warm bath was given him, with stimulant pediluvium, and he was placed upon large doses of quinine and Dover's powder. He died comatose, at 12 o'clock noon, February 28th, without any marked change in the symptoms.

AUTOPSY, 10 O'CLOCK P.M., MARCH 1ST, 1865.

Body not emaciated; rigor mortis not well marked.

*Brain.*—The pia mater, over the whole surface of the cerebrum, was covered with a thick deposition of lymph and pus; brain tissue greatly congested; base of brain covered, in patches, with thick, yellow pus; pons varolii covered with pus in large quantity; brain softened throughout its whole extent, and the convolutions were full of serum and pus; one drachm of bloody serum was found in each lateral ventricle; the membranes of the cord were infiltrated with pus, and some two ounces were collected in a cup on section of the cord through the lower cervical region.

*Chest.*—The lungs were normal. Heart:—Some effusion in the pericardium; heart looks anæmic, with considerable fatty deposit; is enlarged one-quarter, and the tissue is hard and horny to the feel; large albuminous clots were found in the ventricles, the clot being very firm and hard, and each clot passing up into the corresponding auricle; the valves of the aorta were very flabby.

*Abdomen.*—Liver:—Gall-bladder greatly distended; substance of the gland normal. Spleen hypertrophied. Kidneys normal. Intestines:—Omentum very fatty; mesenteric glands enlarged; calibre of the ileum contracted. Bladder greatly distended with decomposing urine; its walls were greatly inflamed and discolored, the mucous lining presenting livid spots.

CASE VI. F. M. Dwyre, American, aged 23, private, Co. C., 9th Maine Vols. Admitted January 15th, 1865, with high fever; pulse 120; severe pain in the back; tongue brown and dry; some epistaxis; urine high colored, with thick phosphatic sediment; eyes injected; pupils contracted; the skin has a jaundiced appearance, and is dry and harsh.

Soon after admission, he became delirious, and so continued, without lucid intervals, until death.

*Progress of case.*—For the first three days, there was retention of urine. The catheter was used. The urine was high colored; had a strong smell, and deposited a heavy, reddish sediment. On the fourth day, the urine became more free, of light color, and passed naturally. The pupils remain contracted; some trismus manifest; also difficulty in deglutition. Fifth day, some jactitation manifest; hearing and vision very obtuse. Patient continued in about the same state, with no other very noticeable symptoms, until January 24th, when he died.

AUTOPSY, 12 O'CLOCK M., JANUARY 25TH, 1865.

Body greatly emaciated; and the skin is very yellow throughout its whole extent. On removing the skull cap, the dura mater was found intensely congested; the pia mater was covered with lymph, with here and there patches of pus; the cerebrum was considerably softened in places; large quantities of pus were found covering the base of the cerebellum, medulla oblongata, and optic tract; the membranes of the cord were distended with serum, some pus also was found in them; the cerebellum was very soft, almost disorganized; the lateral ventricles were full of bloody serum, with pus in the cornua.

*Chest.*—Right lung healthy; left lung found to be undergoing hepatization; lymph was suffused over its entire surface, and adhesions had commenced forming to the pleura costalis. Heart:—Some effusion in the pericardium; organ otherwise normal.

*Abdomen.*—Liver fully one-third larger than natural, and its entire surface beautifully mottled; the gall-bladder was quite empty; substance of liver quite soft, and one could easily push the finger through it; spleen and kidneys normal; the stomach was inflated, also the transverse colon, which was greatly distended, measuring 15 inches in circumference; right colon found adherent to the peritoneum, along its whole tract, and considerably congested; appendix vermiformis enlarged and greatly congested; three inches of the ileum found intussuscepted in one place, and four inches in another. The vessels of the whole intestinal tract were injected; the glands of the mesentery were enlarged; bladder full, and closely adherent to the peritoneum. The adhesions of the colon and bladder were old, very firm and thick, containing an abundance of cellular tissue. (This man had, at no time, been troubled with diarrhœa.)

CASE VII. Joshua J. Drake, private, Co. H., 199th Pa. Vols., aged 26 years, American. Entered hospital January 18th, 1864. At the time of his entrance, he seemed to be suffering from well marked symptoms of typhoid fever. The tongue was dry and brown; teeth encrusted with sordes; pulse full and rapid, 120 in the minute; great sub-sultus tendinum; furious delirium; an occasional cough; pupils natural; urine scanty and high colored.

*Treatment.*—He was bathed and placed in bed, with sinapisms to the feet and cold to the head; Dover's powder and nitrate of potash were followed by a blister to the back of the neck.

*Progress of case.*—The symptoms noticeable on admission did not abate, but continued with an increase of the sub-sultus, and a tendency to opisthotonos; some epistaxis; eyes injected, and coma-like stupor; urine passed off freely, sometimes involuntarily; bowels moderately lax; no change in pupil, and he died January 22d, at 8 o'clock P.M.

AUTOPSY, 1 O'CLOCK P.M., JANUARY 23, 1865.

Body emaciated; some discoloration about the abdomen; the toes of each foot look blue.

*Brain.*—Dura mater highly engorged; some effusion between the pia mater and brain; slight exudation of lymph over the cerebrum and some effusion in the convolutions, with corpuscles of pus contained; substance of cerebrum softened; no effusion found in the ventricles; membranes of the cord distended with serum; cerebellum covered with lymph; slight exudation of pus over the optic tract.

*Chest.*—No effusion in the chest. Left lung:—Upper lobe consolidated; lower lobe highly congested. Right lung shows a highly inflamed condition, and, on cutting into the tissue, pus exudes. Heart:—Pericardium contains eight ounces of serum; slight pericarditis manifest; blood in the ventricles not coagulated; the right auricle contains an albuminous clot.

*Abdomen.*—Liver enlarged one-third; gall-bladder enormously distended; substance of liver apparently healthy; spleen weighs 1½ lbs; kidneys normal; stomach healthy, and contains six ounces of fluid.

*Intestines.*—Calibre of the ileum diminished fully two-thirds; colon filled with fæcal matter; some congestion throughout the whole tract of the jejunum; otherwise, the intestines are healthy. The bladder was distended with urine.

Case VIII. David Small, Co. I., 9th Maine Vols., aged 16 years, American. Admitted to hospital January 18th, 1865. On admission, there was well marked eruption of rubeola; skin hot and dry; pulse 120; some cough; tongue red and dry, especially through the centre; pupils natural; no delirium; respiration very hurried; aud patient seemed stupid.

*Treatment.*—Was bathed, then put to bed, when liq. ammoniæ acetat. was given, together with quinine and milk punch.

*Progress of case.*—No change manifest in symptoms, until the 18th, when the patient became delirious; pupils contracted; tendency to keep the head thrown back. No medicine given after this. Patient died January 23d, at 9:30 o'clock A.M.

SECTIO CADAV. 12 O'CLOCK M., JANUARY 23.

Body extremely emaciated; rigor mortis well marked.

*Brain.*—Membranes seem engorged with blood, and over the whole surface of the cerebrum there is a well marked arterial and venous congestion. Underneath the arachnoid membrane, there is an extensive deposition of lymph, also patches of flocculent pus; the sulci of the brain are filled with serum. Six drachms of serum found in the left lateral ventricle; none found in the right. Cerebellum softened, otherwise presents the same appearance as the cerebrum. Some pus found around the optic commissure. Pons varolii softened.

*Chest.*—Four ounces of serum in the pleural cavity. Right lung:—Lower lobe hepatized; false membrane is forming over the posterior surface. On cutting into the substance of the lower lobe, pus exudes. Left lung normal. Heart:—Pericardium contains four ounces of serum; heart atrophied, and the muscular tissue much softened.

*Abdomen.*—Liver one-third larger than normal; substance greatly congested; gall-bladder atrophied, empty, and congested. Kidneys slightly enlarged, otherwise normal. Spleen normal.

*Intestines.*—Intussusception of ileum for eight inches, old in in character; mucous membrane of the containing part much thickened and congested; whole intestinal tract engorged with blood, but no signs of active peritonitis; stomach enlarged and filled with flatus, also contains four ounces of fluid.

There was phimosis of the penis in this case, and the boy was known as a masturbator. It should have been stated that on removal of the brain, eight ounces of serum escaped through the foramen magnum, and that the medulla oblongata and cord were highly congested. There was also slight adhesion of the pia mater to the brain.

CASE IX. Arthur Smith, aged 23, private, Co. G., 7th Conn. Vols. Admitted to hospital January 6th, 1864. This man was taken with a chill, followed by fever, and complained of pain and soreness throughout his whole body; pupils natural; occasional vomiting of greenish matter.

*Treatment.*—Given pulv. Doveri and quinine, and $\frac{1}{4}$ gr. of morphine at tattoo. The skin seemed normal; and urine passed naturally.

*Progress of case.*—No change in symptoms until just prior to his death, when he became delirious; pupils dilated; and had tonic spasm. He died comatose at 12 M., January 22d.

SECTIO CADAV. 4 P.M., JANUARY 22.

Body slightly emaciated; rigor mortis not well marked.

*Brain.*—Dura mater distended with serum; slight adhesions of the pia mater to the brain; substance of brain greatly congested, with exudation of pus over its whole surface; cerebrum softened; cerebellum so very soft that a stream of water disorganized it; no effusion within the ventricles; spinal cord congested.

*Chest.*—Right lung normal; left lung congested, and the upper lobe throwing out lymph. Heart:—Pericardium contained eight ounces of serum; the blood found in the cavities of the heart was fluid; structure of the heart normal.

*Abdomen.* —Liver hypertrophied; gall-bladder distended. Intestines:—Colon filled with fæcal matter, well digested and of good color; small intestines congested; patches of ulceration found throughout the whole tract of the ileum; spleen somewhat enlarged; kidneys normal; bladder found nearly empty.

[*To be continued.*]

